# School Prejudice and Substance Use from Adolescence to Emerging Adulthood in the United States: Variation across Race and Ethnicity

**DOI:** 10.3390/ijerph20054171

**Published:** 2023-02-25

**Authors:** Xing Zhang, Daniel B. Lee

**Affiliations:** 1 College of Health Solutions, Arizona State University, Phoenix, AZ 85004, USA; 2 Institute for Firearm Injury Prevention, University of Michigan, Ann Arbor, MI 48109, USA

**Keywords:** school prejudice, adolescence, emerging adulthood, substance use, race and ethnicity

## Abstract

Background: Racial and ethnic disparities in health outcomes have been consistently documented in the health literature. Until recently, many studies have evidenced associations between prejudice and health behaviors using cross-sectional data. However, studies assessing the link between school prejudice and health behaviors from adolescence to adulthood are limited. Methods: To address this gap, we use data from Waves I, II, and III of the National Longitudinal Study of Adolescent to Adult Health (1994–2002) to examine how perceptions of school prejudice over time influence cigarette smoking, alcohol use, and marijuana use from adolescence to emerging adulthood. We also examine variation across race and ethnicity. Results: Results indicate that school prejudice in adolescence (Wave I) is associated with higher cigarette use, alcohol use, and marijuana use in later adolescence (Wave II). White and Asian adolescents who perceived school prejudice were more likely to use alcohol, while Hispanic adolescents were more likely to use marijuana. Conclusions: Efforts to reduce school prejudice among adolescents may have implications in reducing substance use.

## 1. Introduction

In 2043, the United States is projected to become a society that predominantly consists of people of color [1]. As racial and ethnic diversity increases, it is important to understand racial boundaries and intergroup relations including prejudice [2]. In addition, as prejudice is a ubiquitous experience in the lives of racial minorities, it is crucial to study prejudice as a pathogenic factor that contributes to racial health disparities, specifically substance use. This study explores whether perceptions of prejudice in school (school prejudice) influences substance use (i.e., cigarette, alcohol, and marijuana use) during adolescence and emerging adulthood [3]. Of note, researchers have pointed out that experiences of school prejudice can predict increased alcohol use and marijuana use [4,5,6]. Adolescence and emerging adulthood are particularly important developmental periods to examine prejudice and substance use as these experiences may negatively implicate health in adulthood [3]. Additionally, prior studies examining school prejudice and substance use have limited their examination to White and Black adolescents [4], which does not reflect the diverse racial and ethnic composition of the United States. Many of these studies have also studied adolescents [4,5] and have not examined the link between prejudice and substance use across developmental periods. This is a gap in the literature as Black adolescents are less likely to use tobacco, marijuana, and alcohol than White adolescents, whereas Black adults are more likely to use these substances than White adults [7]. This study advances our understanding of how school prejudice influences various types of substance use during adolescence and emerging adulthood for Whites, Blacks, Hispanics, and Asians.

Guided by the biopsychosocial model of racism and health [8], we examine the following research questions: how are perceptions of school prejudice associated with substance use from adolescence to emerging adulthood? How do these associations vary by race and ethnicity? Are changes in perceptions of school prejudice (those who perceived increased, decreased, or constant prejudice) associated with substance use?

### 1.1. Theoretical Frameworks

#### 1.1.1. Defining Prejudice

Allport [9] (p. 9) defined prejudice as “an antipathy directed toward a group as a whole, or toward an individual because he is a member of that group.” In further elaboration, Blumer [10] defined four facets of racial prejudice, meant to preserve the position of the dominant group: a feeling of superiority, that the subordinate race is different and threatens the power of the dominant group, and a feeling of entitlement to privilege. Clark et al. [8] (p. 808) conceptualized prejudice as a product of racism and defined perceived racism as “the subjective experience of prejudice or discrimination.” Although prejudice can occur in various socio-ecological contexts (e.g., neighborhood, internet chat rooms), we focus our investigation on school prejudice as adolescents spend a significant proportion of their time at school [11].

#### 1.1.2. Biopsychosocial Model of Racism as a Stressor

The biopsychosocial model of racism and health posits that exposure to racism is a significant source of stress for people in racially minoritized groups and can ultimately contribute to health disparities [8]. Racism-related stressors, such as experiencing prejudice, can tax and deplete coping resources. In turn, chronically perceiving prejudice may contribute to greater alcohol and substance use to mitigate the psychological consequences (e.g., anxiety) of prejudice [8,12,13,14,15].

### 1.2. Prejudice and Substance Use

Previous studies have found that perceived prejudice predicts greater substance use among adolescents [4,16,17,18,19] through the mechanism of coping with racism. However, many studies have either used cross-sectional data or focused their investigation within a single developmental period, with the exception of Goosby and Walsemann [17] and Respress et al. [4]. In line with studies that indicate the importance of examining the prejudice-substance use link across the life course (e.g., cross-over effect in substance use [7]), we have limited knowledge on whether changes in perceptions of prejudice inform changes in substance use. Given the current state of research on racism and substance use, one might surmise that increases in perceptions of prejudice over time are associated with increased substance use, whereas decreases in perceptions of prejudice are associated with decreased substance use. Experiences of school prejudice may remain salient in the transition to adulthood, as emerging adults navigate different social roles and responsibilities.

### 1.3. Hypotheses

Based on prior literature, we test the following hypotheses:

1. School prejudice will be associated with increased substance use in adolescence and emerging adulthood.
2. Increases in perceptions of school prejudice will be associated with a greater likelihood of substance use relative to those who perceive a constant level of school prejudice.
3. Decreases in perceptions of school prejudice will be associated with a decreased likelihood of substance use relative to those who perceive a constant level of school prejudice.
4. Relative to white adolescents, the association between school prejudice and substance use will be larger for adolescents of color.

## 2. Materials and Methods

The National Longitudinal Study of Adolescent to Adult Health (Add Health) was used to answer the research questions [20]. Add Health is a nationally representative, school-based sample of adolescents in the 7th–12th grades based in the United States. Wave I was administered from 1994–1995, Wave II from 1995–1996, and Wave III from 2001–2002. Respondents were 12–19 years old at Wave I, 13–20 years old at Wave II, and 18–28 years old at Wave III. Respondents who were not White, Black, Hispanic or Asian were excluded from the analytic sample due to small sizes. The analytic sample yielded a size of 8,955 respondents, who had no missing data on the primary independent and dependent variables and had valid longitudinal sample weights.

### 2.1. Measures

#### 2.1.1. Dependent Variables

The primary dependent variables at Waves II (adolescence) and III (emerging adulthood) were a range of alcohol, tobacco, and other drug use, including ever smoking, regularly smoking, ever drinking alcohol, and ever smoking marijuana. These variables were examined separately as individual outcomes.

Ever smoked came from the yes or no question “have you ever tried cigarette smoking, even just 1 or 2 puffs?”

Regularly smoked came from the yes or no question “have you ever smoked cigarettes regularly, that is, at least 1 cigarette every day for 30 days?”

Ever drank alcohol came from the yes or no question “since (prior interview year), have you had a drink of beer, wine, or liquor—not just a sip or taste of someone else’s drink—more than two or three times?”

Ever used marijuana can from the yes or no question “since (prior interview year), have you tried or used marijuana?”

#### 2.1.2. Independent Variables

The main predictors of interest included perceptions of prejudice at school measured at Waves I and II.

School prejudice was measured using the following question: “How strongly do you agree or disagree with the following statement? The students at this school are prejudiced”. Answers ranged from strongly disagree (0) to strongly agree (4).

An additional measure of school prejudice, changes in school prejudice, assessed longitudinal changes in perceptions of prejudice from Wave I to II. Three categories were created: perceptions of school prejudice remained the same from Wave I to Wave II (=0), perceptions of school prejudice decreased from Wave I to Wave II (=1), and perceptions of school prejudice increased from Wave I to II (=2).

#### 2.1.3. Control Variables

Controls for prior substance use included ever smoked at Wave I, regular smoking at Wave I, ever drank alcohol at Wave I, and ever using marijuana at Wave I. Background characteristics included the age of the respondent at the time of interview, race and ethnicity, gender, immigrant generation status, maternal education, family structure at Wave I, the quartile of White students at the school the respondent attended at Wave I, and the Aid to Family with Dependent Children (AFDC) status of the respondent at the time of interview.

Age was approximated by subtracting the respondent’s birth year from the interview year.

Race and ethnicity included White, Black, Hispanic, and Asian respondents, and was used from Add Health’s constructed race variable, and whether the respondent identified as Hispanic at Wave I [21].

Immigrant generation status included first, second, and third-generation immigrants, gathered from whether the respondent was born in the United States, and whether the respondent’s parents were born in the United States.

Maternal educational attainment was assessed at Wave I and included the following categories: less than high school, high school, some college, college and more, and do not know.

Family structure at Wave I included four categories of two biological parents, two parents (one or both non-biological), a single parent, and other (Harris, 1999). The quartile of White students at wave I included four categories: 0%, 1–66%, 67–93%, and 94–100%.

AFDC status was whether the respondent received the AFDC at Waves II and III.

### 2.2. Methods

Logistic regression analysis was used to predict the association of perceived school prejudice, changes in perceived school prejudice, and substance use at Waves II and III. Separate analyses were conducted by race and ethnicity. Models with all the controls included are shown. Longitudinal survey weights were used to ensure that the results were nationally representative.

## 3. Results

### 3.1. Descriptive Statistics

#### Overall Sample

Summary statistics for the analytic sample are displayed in Table 1. In adolescence (Wave I), most respondents perceived an average level of prejudice. At Wave II, perceptions of prejudice slightly increased. Approximately 39% of adolescents perceived the same level of school prejudice from Wave I to II, followed by 29% who perceived decreased prejudice and 32% who perceived increased prejudice. Changes in substance use were the largest between Waves II and III; at Wave III, almost 75% of emerging adults had ever smoked a cigarette, 41% regularly smoked a cigarette, 78% drank alcohol, and 52% used marijuana. In terms of individual characteristics, respondents were on average 16 years old at Wave II and 22 years old at Wave III, and 51% female. The majority of the sample was White (69%), and most respondents were born in the United States. Most respondents were first or second-generation immigrants. Family characteristics showed that most respondents’ mothers had completed a high school degree or more and that most respondents had been living in families with two biological parents at Wave I (61%). The majority of respondents, 61%, had attended predominantly White high schools in adolescence.

### 3.2. Differences across Race and Ethnicity

Table 2 shows descriptive statistics of the main predictors and outcomes by race and ethnicity. Perceptions of school prejudice were highest among White adolescents at Waves I and II. Perceptions of increased school prejudice were highest among Asian adolescents; 36% reported increased perceptions of prejudice over time. Cigarette smoking was highest among White adolescents and emerging adults. Alcohol use was highest among White emerging adults; 84% reported drinking alcohol. Marijuana use in adolescence was lowest among Asians, while the highest was among Whites during emerging adulthood.

#### 3.2.1. Analytic Results: School Prejudice and Substance Use

Table 3 (Panel A) shows how school prejudice at Wave I and changes in school prejudice from Waves I to II were associated with substance use in adolescence and emerging adulthood among the full sample. In adolescence, school prejudice experienced at Wave I was associated with higher odds of ever smoked, regular smoking, drinking alcohol, and using marijuana at Wave II.

Table 3 (Panel B) shows how school prejudice at Waves I and II, and changes in school prejudice from Waves I to II, were associated with substance use in emerging adulthood. In emerging adulthood, respondents’ perceptions of school prejudice at Waves I and II, as well as changes in perceptions of school prejudice, were not significantly associated with substance use.

#### 3.2.2. Results Analysis: School Prejudice and Substance Use by Race and Ethnicity among White and Black Respondents

Table 4 (Panel A) shows the associations between school prejudice at Wave I, changes in school prejudice from Waves I and II, and substance use in adolescence. Among White adolescents, those who perceived a decrease in school prejudice from Wave I to II were significantly less likely to ever smoke and regularly smoke cigarettes. However, White adolescents who perceived school prejudice at Wave I had a significantly greater likelihood of ever drinking alcohol by late adolescence. Among Black adolescents, however, school prejudice was not significantly associated with any substance use.

Table 4 (Panel B) shows results from logistic regressions showing associations between school prejudice at Wave I, II, changes in school prejudice from Waves I and II, and substance use in emerging adulthood. In emerging adulthood, school prejudice was not significantly associated with substance use among White adolescents or Black adolescents.

#### 3.2.3. Results Analysis: School Prejudice and Substance Use by Race and Ethnicity among Hispanic and Asian Respondents

Table 5 (Panel A) shows associations between school prejudice at Wave I, changes in school prejudice from Waves I and II, and substance use among Hispanic and Asian adolescents. Among Hispanic adolescents, school prejudice experienced at Wave I was significantly associated with an increased likelihood of marijuana use at Wave II. On the other hand, school prejudice and changes in school prejudice were not significantly associated with other forms of substance use such as ever smoking, regularly smoking, and alcohol use among Hispanic adolescents. Among Asian adolescents, only school prejudice at Wave I was significantly associated with increased odds of ever drinking at Wave II. Changes in school prejudice were not significantly associated with increased odds of substance use among Asian adolescents.

Table 5 (Panel B) shows associations between school prejudice at Wave I, changes in school prejudice from Waves I and II, and substance use among Hispanic and Asian emerging adults. School prejudice at Wave I, changes in school prejudice from Waves I to II, and school prejudice at Wave II were not significantly associated with substance use among Hispanic nor Asian emerging adults.

## 4. Discussion

We found that school prejudice and temporal changes in school prejudice were associated with future substance use during adolescence and emerging adulthood. We also identified racial and ethnic variation in the longitudinal association between school prejudice and substance use outcomes. In the overall sample, we found that school prejudice was significantly associated with increased substance use in adolescence—specifically an increased likelihood of ever smoking, regular smoking, ever drinking alcohol, and ever using marijuana. Importantly, the longitudinal associations between school prejudice and substance use outcomes varied by race and ethnicity. Specifically, among White and Asian adolescents, school prejudice was associated with an increased likelihood of ever drinking alcohol. Among Hispanic adolescents, school prejudice was significantly associated with increased odds of ever using marijuana. Three themes emerged from our study findings: (1) School prejudice was found to be associated with increased substance use, but only during adolescence; (2) Changes in school prejudice over time were significantly associated with substance use; and (3) There was racial and ethnic variation in how school prejudice was associated with substance use.

The first theme that emerged from this study was that school prejudice was associated with increased substance use, but only during adolescence. We hypothesized that school prejudice would be associated with increased substance use in adolescence and emerging adulthood. This finding confirms the biopsychosocial model of racism as a stressor [8], which posits that racism is a stressful experience that can lead to coping behaviors such as substance use. Indeed, we found that adolescents who perceived greater school prejudice were more likely to use alcohol, smoke cigarettes, and use marijuana, confirming findings from previous studies [12,16,17]. However, school prejudice experienced in adolescence was not associated with substance use during emerging adulthood, suggesting that other factors may be more prominent in explaining substance use during emerging adulthood, such as racism-related experiences perpetrated by adults in the school or neighborhood (e.g., law enforcement, teachers), socioeconomic status [22,23], and peer influences [24]. In addition, given that adolescents spend the majority of their time at school, they may be more strongly impacted by the school environment compared to emerging adults, who may be in the labor force [11,25].

The second theme that emerged from our study was that changes in school prejudice across adolescence were associated with substance use in adolescence and emerging adulthood, but only among White adolescents. We expected to find that increases in perceptions of school prejudice would be associated with a greater likelihood of substance use relative to those who perceived a constant level of school prejudice, and that decreases in perceptions of school prejudice would be associated with a decreased likelihood of substance use relative to those who perceived a constant level of school prejudice. Among White adolescents, those who perceived decreases in perceptions of school prejudice were significantly less likely to ever smoke and regularly smoke. Although school prejudice was associated with future substance use for all youth, White adolescents were the most responsive to changes in prejudice because youth of color are aware of and contend with racial prejudice and discrimination as early as childhood [26]. Therefore, changes in school prejudice may be less impactful if youth routinely deal with discrimination and prejudice since childhood. This finding suggests that decreases in perceptions of school prejudice may have positive ramifications for substance use due to a decreased need to cope with a stressful experience [8].

The third theme that emerged from this study was that we found racial and ethnic variation in the association between school prejudice and substance use. We expected that students of color would be more likely to be affected by school prejudice and have greater substance use. Descriptively, we found that White adolescents perceived the highest levels of school prejudice, relative to Black, Hispanic, and Asian adolescents, consistent with prior research using Add Health data from Wave II [27]. However, we found that Hispanic and Asian adolescents had larger magnitudes of associations of school prejudice and substance use relative to White adolescents, particularly for marijuana and alcohol use. This suggests that school prejudice is more strongly associated with substance use among Hispanic and Asian adolescents than White and Black adolescents.

## 5. Limitations

Limitations of this study include the following: First, causal inferences cannot be drawn from the findings. Second, there were limitations to the school prejudice variable. The measure of school prejudice was used as a proxy for experiences of racism and other socially inequitable experiences (e.g., sexism), which were not directly measured in the National Longitudinal Study of Adolescent to Adult Health during adolescence. The measure of school prejudice was a perceived measure of whether other students at school were prejudiced, rather than the adolescent’s own experience of school prejudice. Therefore, the interpretation of the results may not necessarily reflect the individual’s own experience of prejudice, but a school-level perception of other students. Perceptions of school prejudice targeted to a specific racial and ethnic group cannot be inferred. Third, since data was first measured in adolescence, there were no measures of substance use in childhood to be used as control variables. Fourth, due to small sample sizes, we were unable to examine other racial and ethnic groups, such as Native Americans and mixed-race individuals. Fifth, we did not examine the mechanisms linking school prejudice to substance use, such as anxiety and depression.

## 6. Future Research 

Future research should examine the psychological mechanisms linking school prejudice to substance use, including anxiety and depressive symptoms. In addition, examining protective factors of school prejudice and substance use, specifically examining mediators and moderators such as friendship networks, family relationships, after-school activity participation, and neighborhood support can be researched. In addition, variation by gender and socioeconomic status should be explored.

## 7. Conclusions

In conclusion, interventions and public policies aimed at reducing school prejudice may have implications for reducing adolescents’ substance use, including alcohol, marijuana, and cigarettes. These interventions and public policies to reduce school prejudice may be associated with decreased substance use among students of color, particularly Hispanic and Asian adolescents.

## Figures and Tables

**Table 1:** Summary Statistics for the Analytic Sample.

Variable	Mean	SD	Range
School prejudice at Wave I	3.10	0.05	1 to 5
School prejudice at Wave II	3.17	0.05	1 to 5
Change in school prejudice from Wave I to II			0 to 2
Perception of prejudice remained the same	0.39	0.01	
Perception of prejudice decreased from Wave I to II	0.29	0.01	
Perception of prejudice increased from Wave I to II	0.32	0.01	
Ever smoked at Wave I	0.54	0.01	0 to 1
Ever smoked at Wave II	0.45	0.01	
Ever smoked at Wave III	0.75	0.01	
Regularly smoked at Wave I	0.17	0.01	0 to 1
Regularly smoked at Wave II	0.19	0.01	
Regularly smoked at Wave III	0.41	0.01	
Drank alcohol at Wave I	0.51	0.01	0 to 1
Drank alcohol at Wave II	0.48	0.01	
Drank alcohol at Wave III	0.78	0.01	
Used marijuana at Wave I	0.23	0.01	0 to 1
Used marijuana at Wave II	0.24	0.01	
Used marijuana at Wave III	0.52	0.01	
Age of respondent at Wave II	16.37	1.62	13–22
Age of respondent at Wave III	21.60	1.82	18–28
Race/ethnicity			1 to 4
NH White	0.69	0.03	
NH Black	0.15	0.02	
Hispanic	0.12	0.02	
NH Asian	0.04	0.01	
Gender—Female	0.51	0.01	1 to 2
Immigrant generation status			1 to 4
1st generation	0.46	0.02	
2nd generation	0.49	0.01	
3rd generation	0.06	0.01	
Mother’s education at Wave I			1 to 5
Less than HS	0.14	0.01	
HS grad or GED	0.35	0.01	
Some college	0.19	0.01	
Completed college+	0.27	0.02	
Don’t know	0.05	0.00	
Family structure at Wave I			1 to 4
Two biological parents	0.61	0.01	
Two parents (step or bio)	0.16	0.01	
Single parent	0.20	0.01	
Other family arrangement	0.03	0.00	
Quartile White in School			1 to 4
0%	0.09	0.02	
1–66%	0.29	0.04	
67–93%	0.33	0.04	
94–100%	0.28	0.04	
*n*	8955		

**Table 2:** Descriptives of the means for the main predictors and outcomes by race and ethnicity.

	NH White	NH Black	Hispanic	NH Asian
School prejudice at Wave I	3.25	3.41 ^a^	2.95 ^b^	2.93 ^c^
School prejudice at Wave II	3.31	2.59 ^a^	3.05 ^b^	3.03 ^c^
Changes in school prejudice from Wave I to Wave II				
Perception of prejudice remained the same	0.39	0.42	0.39	0.33
Prejudice decreased	0.29	0.28	0.29	0.30
Prejudice increased	0.32	0.30	0.32	0.36
Ever smoked at Wave II	0.49	0.29 ^a^	0.42 ^b^	0.32 ^c^
Ever smoked at Wave III	0.79	0.61 ^a^	0.73 ^b^	0.67 ^c^
Regularly smoking in Wave II	0.23	0.08 ^a^	0.15 ^b^	0.10 ^c^
Regularly smoking at Wave III	0.47	0.23 ^a^	0.32 ^b^	0.31 ^c^
Drank alcohol at Wave II	0.51	0.35 ^a^	0.51	0.34 ^c^
Drank alcohol at Wave III	0.84	0.58 ^a^	0.76 ^b^	0.69 ^c^
Used marijuana at Wave II	0.24	0.22	0.27	0.17 ^c^
Used marijuana at Wave III	0.55	0.42 ^a^	0.50	0.39 ^c^
*n*	8955			

^a^—Black–White differences; ^b^—Hispanic–White differences; ^c^—Asian–White differences, significant at *p* < 0.05.

**Table 3:** Likelihood of substance use for the full sample in Waves II and III.

Panel A. Wave II—Adolescence	Overall Sample											
Variables	Ever Smoked			Regular Smoking			Alcohol			Marijuana		
	B		OR	B		OR	B		OR	B		OR
School prejudice at Wave I	0.07	*	1.07	0.10	**	1.11	0.08	**	1.08	0.09	*	1.09
	(0.04)			(0.04)			(0.03)			(0.04)		
Changes in school prejudice from Wave I to II—Decreased (rel. to no change)	−0.12		0.89	−0.20		0.82	−0.10		0.90	−0.14		0.87
	(0.09)			(0.10)			(0.09)			(0.09)		
Increased	0.04		1.04	0.15		1.16	0.09		1.09	0.10		1.11
	(0.09)			(0.14)			(0.09)			(0.10)		
*n*	8955											
**Panel B. Wave III—Emerging Adulthood**	**Overall Sample**											
**Variables**	**Ever Smoked**			**Regular Smoking**			**Alcohol**			**Marijuana**		
	**B**		**OR**	**B**		**OR**	**B**		**OR**	**B**		
School prejudice at Wave I	−0.04		0.96	0.01		1.01	0.00		1.00	−0.02		0.98
	(0.07)			(0.07)			(0.07)			(0.07)		
Changes in school prejudice from Wave I to II—Decreased (rel. to no change)	0.03		1.03	0.00		1.00	0.01		1.01	0.07		1.07
	(0.13)			(0.13)			(0.12)			(0.11)		
Increased	−0.17		0.84	0.06		1.06	−0.04		0.96	0.03		1.03
	(0.12)			(0.11)			(0.14)			(0.11)		
School prejudice at Wave II	0.04		1.04	0.01		1.01	0.04		1.04	0.06		1.06
	(0.07)			(0.07)			(0.07)			(0.06)		
*n*	8955											

Regular smoking refers to having smoked at least 1 cigarette in the past 30 days; alcohol refers to ever having consumed alcohol more than two times in the past year; marijuana refers to marijuana use in the past year. Standard errors are in parentheses. Controls include age, race and ethnicity, gender, maternal education at Wave I, immigrant generation status, family structure at Wave I, the percentage of White students at the school attended, AFDC status at Waves I and II, ever smoked at Wave I, regularly smoked at Wave I, and ever drank alcohol at Wave I. Controls for the Wave III analyses also included ever smoked at Wave II, regularly smoked at Wave II, and ever drank alcohol at Wave II. * *p* < 0.05 ** *p* < 0.01.

**Table 4:** Likelihood of substance use among White and Black adolescents at Waves II and III.

**Panel A. Wave II—Adolescence**	**NH White Adolescents**								**NH Black Adolescents**							
**Variables**	**Ever Smoked**		**Regular Smoking**		**Alcohol**		**Marijuana**		**Ever Smoked**		**Regular Smoking**		**Alcohol**		**Marijuana**	
	**B**	**OR**	**B**	**OR**	**B**	**OR**	**B**	**OR**	**B**	**OR**	**B**	**OR**	**B**	**OR**	**B**	**OR**
School prejudice at Wave I	0.06	1.06	0.08	1.08	0.11 **	1.12	0.09	1.09	0.02	1.02	0.00	1.00	−0.04	0.96	−0.07	0.93
	(0.05)		(0.05)		(0.04)		(0.06)		(0.08)		(0.14)		(0.07)		(0.11)	
Changes in school prejudice from Wave I to II—Decreased (rel. to no change)	−0.23 *	0.79	−0.30 *	0.74	−0.09	0.91	−0.18	0.84	0.33	1.39	−0.25	0.78	−0.13	0.88	0.05	1.05
	(0.11)		(0.13)		(0.11)		(0.11)		(0.18)		(0.34)		(0.20)		(0.26)	
Increased	0.06	1.06	0.11	1.12	0.12	1.13	0.10	1.11	−0.08	0.92	0.18	1.20	−0.04	0.96	−0.02	0.98
	(0.11)		(0.12)		(0.11)		(0.12)		(0.20)		(0.29)		(0.14)		(0.24)	
*n*	5046								1872							
**Panel B. Wave III—Emerging Adulthood**	**NH White Emerging Adults**								**NH Black Emerging Adults**							
**Variables**	**Ever Smoked**		**Regular Smoking**		**Alcohol**		**Marijuana**		**Ever Smoked**		**Regular Smoking**		**Alcohol**		**Marijuana**	
	**B**	**OR**	**B**	**OR**	**B**	**OR**	**B**	**OR**	**B**	**OR**	**B**	**OR**	**B**	**OR**	**B**	**OR**
School prejudice at Wave I	−0.06	0.94	−0.07	0.93	−0.03	0.97	−0.06	0.94	0.09	1.09	0.07	1.07	0.06	1.06	0.12	1.13
	(0.08)		(0.08)		(0.10)	0.90	(0.08)		(0.13)		(0.19)		(0.11)		(0.11)	
Changes in school prejudice from Wave I to II—Decreased (rel. to no change)	0.10	1.11	0.14	1.15	0.11	1.12	0.10	1.11	−0.24	0.79	−0.02	0.98	−0.11	0.90	0.03	1.03
	(0.16)		(0.16)		(0.15)		(0.14)		(0.24)		(0.35)		(0.30)		(0.23)	
Increased	−0.22	0.80	0.03	1.03	−0.01	0.99	0.01	1.01	−0.03	0.97	0.18	1.20	−0.17	0.84	0.07	1.07
	(0.15)		(0.14)		(0.18)		(0.12)		(0.27)		(0.30)		(0.20)		(0.22)	
School prejudice at Wave II	0.08	1.08	0.08	1.08	−0.01	0.99	0.11	1.12	−0.06	0.94	−0.12	0.89	0.05	1.05	−0.14	0.87
	(0.08)		(0.09)		(0.18)		(0.07)		(0.12)		(0.16)		(0.11)		(0.12)	
*n*	5046								1872							

Regular smoking refers to having smoked at least 1 cigarette in the past 30 days; alcohol refers to ever having consumed alcohol more than two times in the past year; marijuana refers to marijuana use in the past year. Standard errors are in parentheses. Controls include age, gender, maternal education at Wave I, immigrant generation status, family structure at Wave I, the percentage of white students at the school attended, AFDC status at Waves I and II, ever smoked at Wave I, regularly smoked at Wave I, and ever drank alcohol at Wave I. Controls for the Wave III analyses also included ever smoking at Wave II, regularly smoked at Wave II, and ever drank alcohol at Wave II. * *p* < 0.05 ** *p* < 0.01.

**Table 5:** Logistic regressions predicting likelihood of substance use for Hispanic and Asian adolescents at Waves II and III.

Panel A. Wave II—Adolescence	Hispanic Adolescents								NH Asian Adolescents							
Variables	Ever Smoked		Regular Smoking		Alcohol		Marijuana		Ever Smoked		Regular Smoking		Alcohol		Marijuana	
	B	OR	B	OR	B	OR	B	OR	B	OR	B	OR	B	OR	B	OR
School prejudice at Wave I	0.08	1.08	0.13	1.14	0.04	1.04	0.20 *	1.22	−0.07	0.93	0.14	1.15	0.35 *	1.42	0.19	1.21
	(0.09)		(0.28)		(0.09)		(0.10)		(0.16)		(0.18)		(0.15)		(0.19)	
Changes in school prejudice from Wave I to II—Decreased (rel. to no change)	−0.04	0.96	0.54	1.72	0.01	1.01	−0.07	0.93	0.33	1.39	0.52	1.68	−0.18	0.84	0.45	1.57
	(0.23)		(0.32)		(0.24)		(0.23)		(0.48)		(0.58)		(0.34)		(0.58)	
Increased	0.28	1.32	0.37	1.45	0.29	1.34	0.32	1.38	−0.46	0.63	0.06	1.06	−0.29	0.75	0.35	1.42
	(0.27)		(0.35)		(0.25)		(0.24)		(0.35)		(0.59)		(0.32)		(0.44)	
*n*	1397								640							
**Panel B. Wave III—Emerging Adulthood**	**Hispanic Adolescents**								**NH Asian Adolescents**							
**Variables**	**Ever Smoked**		**Regular Smoking**		**Alcohol**		**Marijuana**		**Ever Smoked**		**Regular Smoking**		**Alcohol**		**Marijuana**	
	**B**	**OR**	**B**	**OR**	**B**	**OR**	**B**	**OR**	**B**	**OR**	**B**	**OR**	**B**	**OR**	**B**	**OR**
School prejudice at Wave I	−0.12	0.89	0.32	1.38	0.12	1.13	0.03	1.03	−0.01	0.99	0.17	1.19	−0.50	0.61	−0.20	0.82
	(0.20)		(0.21)		(0.17)		(0.18)		(0.26)		(0.41)		(0.29)		(0.30)	
Changes in school prejudice from Wave I to II—Decreased (rel. to no change)	0.02	1.02	−0.62	0.54	−0.37	0.69	0.02	1.02	0.54	1.72	−0.75	0.47	0.35	1.42	0.71	2.03
	(0.38)		(0.36)		(0.34)		(0.16)		(0.56)		(0.82)		(0.43)		(0.50)	
Increased	−0.20	0.82	0.40	1.49	0.26	1.30	−0.14	0.87	0.29	1.34	−0.43	0.65	−0.54	0.58	−0.09	0.91
	(0.36)		(0.36)		(0.38)		(0.31)		(0.55)		(0.62)		(0.67)		(0.47)	
School prejudice at Wave II	−0.20	0.82	−0.31	0.73	−0.21	0.81	0.18	1.20	0.23	1.26	−0.17	0.84	0.40	1.49	0.16	1.17
	(0.36)		(0.20)		(0.18)		(0.34)		(0.27)		(0.37)		(0.28)		(0.27)	
*n*	1397								640							

Regular smoking refers to having smoked at least 1 cigarette in the past 30 days; alcohol refers to ever having consumed alcohol more than two times in the past year; marijuana refers to marijuana use in the past year. Standard errors are in parentheses. Controls include age, gender, maternal education at Wave I, immigrant generation status, family structure at Wave I, the percentage of White students at the school attended, AFDC status at Waves I and II, ever smoked at Wave I, regularly smoked at Wave I, and ever drank alcohol at Wave I. Controls for the Wave III analyses also included ever smoked at wave II, regularly smoked at Wave II, and ever drank alcohol at Wave II. * *p* < 0.05.

## Data Availability

The authors used the restricted use Add Health dataset. Information on access to Add Health is here: https://addhealth.cpc.unc.edu/data/ (accessed on 20 February 2023).
